# Videos about older adults on TikTok

**DOI:** 10.1371/journal.pone.0285987

**Published:** 2023-08-02

**Authors:** Reuben Ng, Nicole Indran

**Affiliations:** 1 Lee Kuan Yew School of Public Policy, National University of Singapore, Singapore, Singapore; 2 Lloyd’s Register Foundation Institute for the Public Understanding of Risk, National University of Singapore, Singapore, Singapore; University of Albany, State University of New York, UNITED STATES

## Abstract

Besides being one of the fastest growing platforms since entering the social media fray in 2016, TikTok is notably monopolized by teenagers, which makes it a veritable source of information not to be overlooked by gerontologists. Currently, most studies regarding age stereotypes on social media have examined content on Twitter and Facebook. Our study explores how older adults are portrayed on TikTok and the factors associated with these portrayals. We analyzed 673 videos with the hashtags #Boomer and/or #OkBoomer that received over 5.4 billion views and categorized them into nine topics. Five of these topics (e.g., ’Warmth/Coldness’) were extracted from previous studies on age stereotypes. The remaining four topics were unique to our dataset (e.g., ‘Wealth Gap’). The outcome variable was ‘Negative Age Stereotypes’ which was rated on a binary scale. One in two videos about older adults featured negative content. As hypothesized, videos containing negative age stereotypes were more likely to be about the ‘Values and Beliefs of Older Adults’ (7 times), ‘Negative Encounters with Older Adults’ (8 times) or ‘Older Adults Antagonizing the Young’ (13 times). Conversely, videos which portrayed older adults as ‘Warm’ were 43% less likely to contain negative stereotypes. As the phenomenon of an aging population fast unfolds, it is imperative that society relinquishes its tendency to stereotype individuals on the grounds of age. By examining the possible mechanisms driving negative stereotypes of older adults on TikTok, our study provides the basis upon which such stereotypes can be counteracted. In doing so, it paves the way both to improve the well-being of older persons and to foster intergenerational solidarity.

## Introduction

In its initial coinage, the term ‘Baby Boomer’ referred to those born between the early 1940s to the early 1960s, when the United States experienced a spike in the number of births after World War II. Today, the generational label has taken on a new meaning. Described by some as ‘Boomer Blaming’ [1], Baby Boomers are frequently pinpointed in public discourse as responsible for a bevy of social and economic issues [2]. In 2019, the phrase ‘Ok, Boomer’ went viral on social media, reflecting widespread beliefs among younger people that Baby Boomers are hindrances to societal progress [3]. By 2030, all Baby Boomers would be at least 65 years of age [4]. To bridge the generational divide and reap the benefits of intergenerational interaction [5], it is first necessary to determine the factors linked to negative stereotypes of older adults. Our study sheds light on this topic by analyzing videos with the hashtags #OkBoomer and/or #Boomer on TikTok.

### Significance of study and hypotheses

Conceptually, this study is significant in that it is among the first to explore stereotypes of older adults on TikTok. Besides being one of the fastest growing platforms since entering the social media fray in 2016 [6]—the application took merely five years to hit a billion monthly active users [7]—TikTok is notably monopolized by teenagers [8], which makes it a veritable source of information not to be overlooked by gerontologists. Furthermore, age stereotypes are conventionally measured through surveys or questionnaires [9]. However, an analysis of videos may present nuances not captured by regular survey-based methods. In addition, older persons have recently begun embracing TikTok [10, 11]. As the usage of social media can affect the well-being and quality of life of older persons [12–14], it is vital to understand how members of this cohort are being portrayed by their younger contemporaries on TikTok. From a practical perspective, this study provides insights into the mechanisms linked to negative stereotypes of older persons, which will in turn lay the foundation for improving their well-being as well as for cultivating intergenerational solidarity.

Existing literature on the way older persons are stereotyped on social media has looked primarily at Facebook [15], Twitter [16–19] and Weibo [20], with only a handful mining content on TikTok. A content analysis of descriptions of publicly accessible Facebook groups about older people discovered that negative age stereotypes predominated [15]. Ageism was especially apparent on Twitter during the COVID-19 crisis [16–19, 21], though calls for intergenerational solidarity were also observed on the application [17, 18]. A thematic analysis of posts uploaded on Weibo—a Chinese microblogging site with features that resemble those on Twitter—revealed that during the pandemic, older people were constructed as warm and competent individuals who actively exercised their agency [20].

Research in gerontology that centers on TikTok revolves largely around the use of the application among older adults [10, 11]. Only one study has examined how older persons are stereotyped by younger people on the platform [22]. This study unpacked videos on TikTok that featured younger people expressing a sense of hostility towards Baby Boomers [22]. Our study differs from the aforementioned in two key aspects. First, unlike the previous study which employed a qualitative approach, our study is quantitative in nature. This allows for a larger number of videos to be analyzed, which will in turn facilitate a more comprehensive understanding of the portrayal of older adults on TikTok. Second, while the previous study focused solely on videos where younger people evaluated Baby Boomers negatively, the current study analyzes videos with and without negative age stereotypes. Hence, our study lends a broader and more nuanced perspective on how older adults are viewed by younger people on TikTok.

Since Butler’s [23] pioneering work on ageism, age stereotypes have become a major subject of gerontological interest. Although negative views dominate the image of older people [24–27], it should be noted that stereotypes of them are not wholly negative. Instead, the broad category of the older person is highly complex and differentiated into multiple subcategories [28, 29]. Positive stereotypes include being warm, generous and kind [30], while negative stereotypes include being slow, irrelevant and incompetent [24]. Recent studies have shown that age stereotypes in the United States have become more negative over the last two centuries [31, 32]—an alarming trend given that the assimilation of negative age stereotypes into one’s self-concept may adversely impact one’s health [33].

Various theories at the micro, meso and macro levels have been proffered to uncover the etiology of ageism [34]. One of these is social identity theory. Social identity refers to the aspects of one’s self-concept that are derived from one’s membership in a particular social group [35, 36]. At the core of social identity theory is the idea that individuals categorize themselves on the basis of perceived similarities and differences. In order to maintain a positive social identity, people engage in intergroup evaluations that favor the ingroup and discriminate against the outgroup [35, 36]. Individuals may also express negative attitudes towards outgroup members when the value of an ingroup is attacked [37]. Evidence has shown that people tend to denigrate younger generations [38]. Thus, we hypothesize that content about ‘Older Adults Antagonizing the Young’ will be linked to negative stereotypes of the older population (Hypothesis 1a).

Another perspective commonly used to account for ageism is that of intergroup threat theory. The theory proposes that prejudice towards particular social groups is rooted in the perception that these groups pose two kinds of threat: realistic and symbolic [39]. Realistic threats refer to threats to the group’s power, resources and welfare, while symbolic threats are threats to one’s worldview, belief system and values. Since Baby Boomers are frequently deemed to be obstructing the more progressive ideologies of the younger generation [3], we hypothesize that ‘Values and Beliefs of Older Adults’ will be linked to negative stereotypes of this population (Hypothesis 1b). Intergroup threat theory further postulates that experiences involving negative contact are a possible antecedent of perceptions of threat. Given the potential for negative intergroup contact to increase prejudice against outgroups [40], we hypothesize that videos concerning ‘Negative Encounters with Older Adults’ will be associated with negative age stereotypes (Hypothesis 1c).

Stereotype content model predicates that the stereotypes applied to social groups fall along two dimensions: warmth and competence [41]. Seen as occupying a low-status and non-competitive social position, the older demographic is typically perceived as a pitied group composed of warm but incompetent individuals [24, 41]. Hence, we hypothesize that content portraying older adults as ‘Warm’ will lessen the chances of negative stereotyping (Hypothesis 2).

In undertaking this study, some important points must be raised. Although interrelated, ‘age’ and ‘generation’ are conceptually distinct [42]. Mannheim [43] defined a generation as a group of people born within a specific period and whose worldviews distinguish them from other generations. According to Mannheim, one’s generational consciousness arises not simply from being part of a certain age cohort, but from the experiences that emerge from being subject to a specific set of social, cultural and historical conditions [44]. Our intention in using the hashtags #Boomer and #OkBoomer as a vehicle for understanding stereotypes of older persons is not to conflate age and generation. Rather, it is motivated by the possibility that many people, including the users creating these TikTok videos, are not actually cognizant of the official range of birth years—as defined by demographers—of Baby Boomers. Today, the term ‘generation’ is often used interchangeably with age [45]. The chances are therefore high that some younger people treat ‘Baby Boomers’ as a catch-all term for older adults.

Additionally, the use of generational categories will inevitably have ramifications on how society makes sense of age and age-related matters [45]. In fact, advocates have recently urged for the use of generational framing to be abolished [46]. In an ageist society, generational framing legitimizes segregation on the basis of age and foments ageism [46]. Moreover, while the phrase ‘Ok, Boomer’ may be a criticism of unprogressive mindsets rather than old age [47], we argue that the phrase ultimately paints older adults as problematic by singling out a particular group of individuals. We therefore analyze videos with the hashtags #Boomer and/or #OkBoomer as a way to understand younger people’s stereotypes of older adults, either in general or those from the Baby Boomer generation. In this study, we use the terms ‘young’ and ‘younger’ to refer to Millennials and members of Generation Z. ‘Old’ and ‘older’ are used to refer to users aged 57—the age of the youngest Baby Boomer in 2021 when the data were collected—and above.

## Methods

### Dataset

Similar to earlier research [48], a new TikTok account was created to collate the videos. This was done to reduce bias since videos on the application are sorted based on a complex algorithm which takes into account the popularity of the post (measured by views, likes, comments and shares), the popularity of the creator (measured by followers and engagement), any previous content that was engaged with, as well as the geographical location of the device used to access the application [48]. No content was previously engaged with to guarantee a common user’s experience in navigating the platform [48].

We compiled publicly available videos (*N* = 1,937) catalogued under the hashtags #Boomer and #OkBoomer which garnered a total of 5.4 billion views. Videos were selected based on the following criteria: (1) Video was publicly available and created by a younger person i.e., videos created by older adults were excluded; (2) Video was in English; (3) Video featured content that was relevant to the hashtag i.e., video should be about older adults. All videos had a maximum duration of one minute as mandated by the platform during the period of the study.

**Fig 1:**
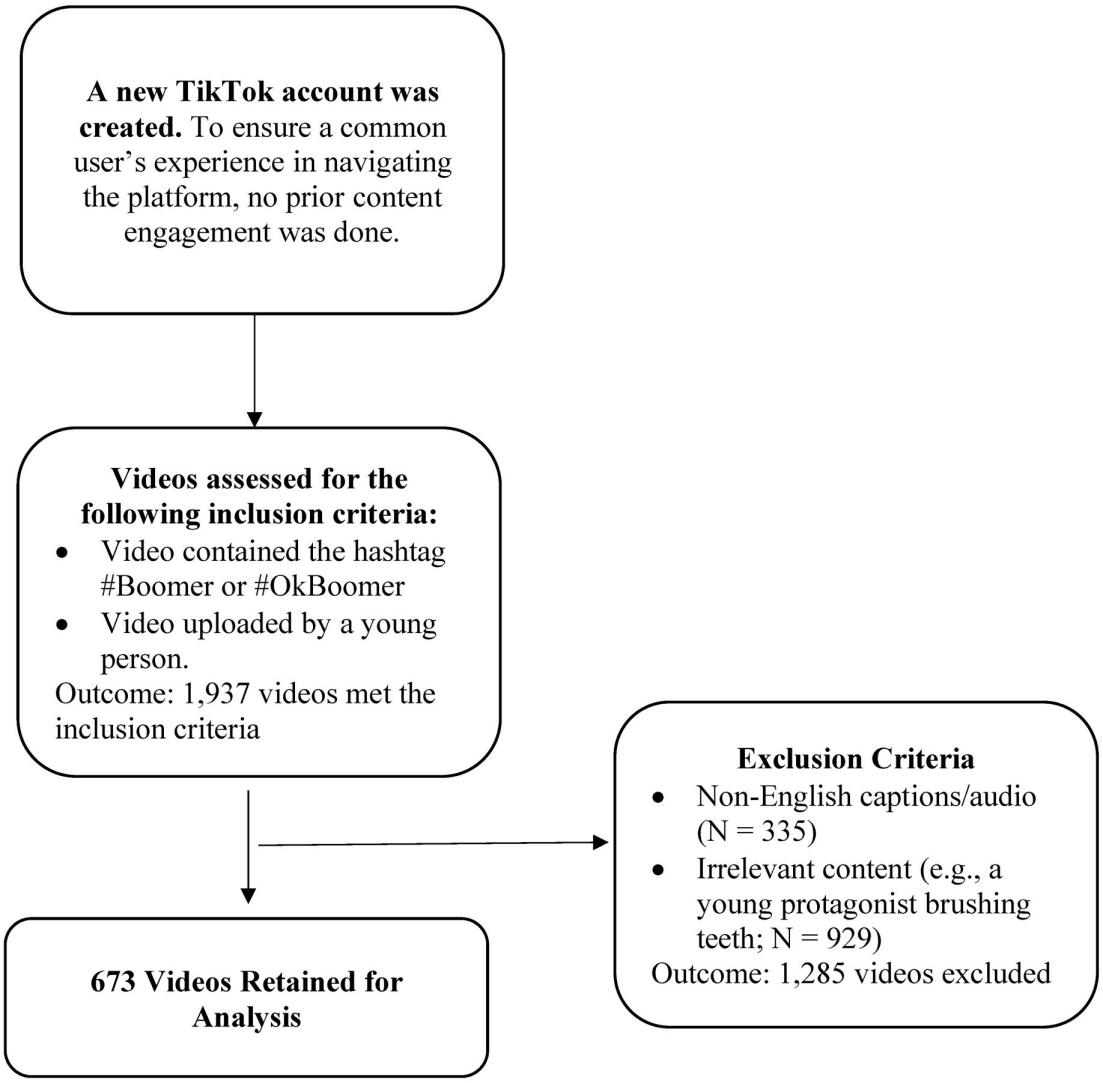
Process of collating TikTok videos created by young people about older adults.

To meet the first inclusion criterion, we processed users’ video frames via Microsoft Azure’s Face API [49]. The user’s profile picture was used in instances where the user’s face was not featured in the video. Microsoft Azure’s Face API extracts information regarding age and has been used in prior research on TikTok [50, 51]. In this study, ‘young’ and ‘younger’ were used to refer to Millennials and members of Generation Z. The maximum age of a ‘younger’ user was 40—the age of the oldest Millennial in 2021 when the data were collected—and due to ethical considerations, the minimum age was set as 16. We used ‘old’ and ‘older’ to refer to users aged 57—the age of the youngest Baby Boomer in 2021—and above. After applying both the inclusion and exclusion criteria, 673 videos were retained for analysis. Fig 1 depicts a flowchart of the data collection process.

### Ethical considerations

As the videos analyzed in our study were publicly available, we did not seek ethical approval or consent from the users whose posts were included in our dataset. However, we recognize that using publicly available data still carries ethical and privacy considerations [52]. Thus, we took steps to protect the privacy of all users whose videos were included in our study, such as by removing any identifying information. We also emphasize that our study does not provide any detailed descriptions of the content of the videos, which means it is difficult to trace the people who posted the videos. The collection of videos and method of analysis complied with the terms and conditions of TikTok.

### Coding of predictors

Following previous research [53, 54], the codebook (Table 1) was developed through both inductive and deductive approaches [55]. In inductive content analyses, codes are derived directly from the data [56]. Meanwhile, analyses led by a directed or deductive approach begin with the identification of an initial set of codes based on past scholarship [56]. We adopted both inductive and deductive approaches to ensure that certain pertinent assumptions informed the analysis while also mindful that new categories would emerge inductively [55].

**Table 1:** Codebook for videos created by younger people about older adults on TikTok.

Variable	Description	Coding
**Outcome**		
Negative Age Stereotypes	Whether attitudes displayed by the person in the video reflect any negative stereotyping of older adults.	0 –Absent 1 –Present
**Predictors Coded using Top-down Approach** (Variables collated from validated scales measuring age stereotypes and/or studies regarding perceptions of aging [29, 31]		
Physical Functioning	Protagonist comments on the physical abilities of an older adult.	0 –Absent 1 –Present
Appearance	Protagonist comments on the appearance of an older adult.	0 –Absent 1 –Present
Illness/Death	Protagonist talks about an older person in relation to illness or death.	0 –Absent 1 –Present
Warmth	Protagonist describes an older adult as very cold or very warm.	1 –Very cold 5 –Very warm
Competence	Protagonist describes an older adult as very incompetent or very competent (Fiske, 2018).	1 –Very incompetent 5 –Very competent
**Predictors Coded using Bottom-up Approach** (Topics that surfaced prominently across multiple videos and that could not be appropriately coded into variables identified using the top-down approach)		
Wealth Gap	Protagonist comments on the existence of a wealth gap between older and younger generations.	0 –Absent 1 –Present
Values and Beliefs of Older Adults	Protagonist describes older adults as having beliefs that either violate or uphold cherished values and norms, whether religious, political or social.	0 –Absent 1 –Present
Negative Encounters with Older Adults	Protagonist recounts a prior negative experience with an older adult.	0 –Absent 1 –Present
Older Adults Antagonizing the Young	Protagonist reacts to a derogatory comment about younger people made by an older adult.	0 –Absent 1 –Present

To develop a preliminary codebook, we first identified a set of categories based on past literature. These variables were collated from validated scales measuring age stereotypes as well as studies regarding perceptions of aging [29, 31]: (1) ‘Physical Functioning’ (e.g., protagonist comments on the physical abilities of an older person); (2) ‘Appearance’ (e.g., protagonist comments on the appearance of an older person); (3) ‘Illness/Death’; (4) ‘Warmth’ (i.e. whether the older person is viewed as warm or cold) [57]; (5) ‘Competence’ (e.g., whether the older person is viewed as competent or incompetent).

The content analysis was subsequently conducted in several stages, with each video viewed twice by two researchers trained in gerontology to ensure familiarity with and immersion in the data [56]. The goal of the first viewing was to confirm the validity of the initial set of categories, as well as to generate codes systematically across the entire dataset. Each researcher modified the codebook independently until all variables were refined and clearly defined. During the first viewing, a new category was added whenever a video featured a particular attribute which could not be suitably coded into any of the existing categories and which was recurrent in the data. During the second viewing, the two coders had frequent discussions during which any discrepancies were reviewed and adjudicated to ensure rigor in the analysis. At this point, the two coders discussed what the codes meant, ensured the relevance of the codes to the research question and identified areas of significant overlap so as to finalize the codebook.

Topics that surfaced prominently across multiple videos and that could not be appropriately coded into the aforementioned categories include: (6) ‘Wealth Gap’ (e.g., protagonist comments on the existence of a wealth gap between older and younger generations); (7) ‘Values and Beliefs of Older Adults’ (i.e., protagonist describes older adults as having beliefs that either violate or uphold cherished values and norms); (8) ‘Negative Encounters with Older Adults’ (e.g., protagonist describes an unpleasant encounter with an older adult); (9) ‘Older Adults Antagonizing the Young’ (e.g., protagonist reacts to negative evaluations of younger people by older adults). With the exception of the ‘Warm/Cold’ and ‘Competent/Incompetent’ variables (Fiske, 2018), both of which were rated on a 5-point scale from 1 (very cold/incompetent) to 5 (very warm/competent), all variables were rated on a binary scale, with the presence of the attribute in the video rated as 1 and the absence of it rated as 0.

### Coding of outcome variable

The outcome variable was ‘Negative Age Stereotypes’. The presence of negative stereotypes was rated 1 and the absence of it—regardless of whether the stereotype was positive or neutral—was rated 0. An example of a video rated 1 is one that featured a younger person saying that older adults have pillaged the economy. An example of a video rated 0 is one that described older adults as being kind. Inter-rater reliability was estimated using weighted Cohen’s kappa for the outcome variable. The percentage agreement between two coders was 97.5% with a weighted Cohen’s kappa of 0.94 (*p* < .001), indicating high inter-rater reliability.

### Analytic strategy

We performed logistic regressions to test our hypotheses. Model 1 included variables extracted from previous studies on age stereotypes derived from a top-down approach: ‘Physical Functioning’, ‘Appearance’, ‘Illness/Death’, ‘Warmth’ and ‘Competence’. Model 2 consisted of variables from Model 1 as well as variables derived from a bottom-up approach: ‘Wealth Gap’, ‘Values and Beliefs of Older Adults’, ‘Negative Encounters with Older Adults’ and ‘Older Adults Antagonizing the Young.’ Analyses were conducted using R version 4.0.3.

## Results

### Descriptive statistics

Overall, 1 in 2 videos (49.3%) contained negative age stereotypes. Of these videos, 79% were related to ‘Negative Encounters with Older Adults’, which is more than 4 times the number for videos without negative age stereotypes. About 58% of the videos included criticisms of the ‘Values and Beliefs of Older Adults’, which is also more than 4 times the number for posts without negative age stereotypes. About 40% featured ‘Older Adults Antagonizing the Young’, which was 18 times more than videos without negative age stereotypes. Negatively rated videos contained more stereotypes of older adults as ‘Cold’. See Table 2 for a summary.

**Table 2:** Description of topics based on whether videos created by younger people about older adults on TikTok include negative age stereotypes.

Topic		Negative Age Stereotypes		p^b^
		Absent (*N* = 341)	Present (*N* = 332)	
Physical Functioning	Absent	331 (97.1%)	331 (99.7%)	0.02
	Present	10 (2.9%)	1 (0.3%)	
Appearance	Absent	334 (97.9%)	327 (98.5%)	0.81
	Present	7 (2.1%)	5 (1.5%)	
Illness/Death	Absent	323 (94.7%)	314 (94.6%)	1.00
	Present	18 (5.3%)	18 (5.4%)	
Warmth		2.63 (1.05)	1.39 (0.76)	0.00
Competence		2.82 (0.63)	2.92 (0.39)	0.01
Wealth Gap	Absent	335 (98.2%)	309 (93.1%)	0.00
	Present	6 (1.8%)	23 (6.9%)	
Values and Beliefs of Older Adults	Absent	293 (85.9%)	140 (42.2%)	0.00
	Present	48 (14.1%)	192 (57.8%)	
Negative Encounters with Older Adults	Absent	282 (82.7%)	70 (21.1%)	0.00
	Present	59 (17.3%)	262 (78.9%)	
Older Adults Antagonizing the Young	Absent	334 (97.9%)	202 (60.8%)	0.00
	Present	7 (2.1%)	130 (39.2%)	

^a^ Table values are N and column % for respective variables.

^b^ P-value is for t-test (continuous variables) or χ2 test (categorical variables).

### Logistic regression of factors associated with negative age stereotypes on TikTok

As hypothesized, videos criticizing the ‘Values and Beliefs of Older Adults’ were 7 times more likely to feature negative age stereotypes (*p* < .001). Videos about ‘Negative Encounters with Older Adults’ were 8 times more likely to contain evidence of negative stereotypes of older adults (*p* < .001). Content about ‘Older Adults Antagonizing the Young’ was 13 times more likely to contain negative age stereotypes (*p* < .001), controlling for other factors (Table 3). Taken together, these findings provide support for hypotheses 1a through 1c. Meanwhile, videos portraying older adults as ‘Warm’ were 43% less likely to contain negative stereotypes, providing support for Hypothesis 2. There was no evidence of multicollinearity as the variance inflation factor (VIF) scores for all variables were below the conservative threshold of 5.

**Table 3:** Logistic regression to analyze topics linked to negative age stereotypes in videos created by younger people about older adults on TikTok.

Model	(1)		(2)	
	Odds Ratio	95% CI	Odds Ratio	95% CI
Physical Functioning	0.31	(0.01–2.52)	0.12	(0.00–4.67)
Appearance	0.68	(0.17–2.54)	0.48	(0.08–2.60)
Illness/Death	1.56	(0.68–3.56)	1.48	(0.46–4.47)
Warmth	0.27***	(0.22–0.33)	0.57***	(0.44–0.74)
Competence	1.08	(0.74–1.60)	1.44	(0.87–2.47)
Wealth Gap			2.90	(0.88–10.16)
Values and Beliefs of Older Adults			7.18***	(4.29–12.36)
Negative Encounters with Older Adults			8.17***	(4.72–14.48)
Older Adults Antagonizing the Young			12.96***	(5.57–34.67)
*N*:	673		673	
AIC:	702.72		496.85	
Pseudo R^2^:	0.26		0.49	

Note:

* *p* < 0.05,

** *p* < 0.01,

*** *p* < 0.001.

Constant not shown.

## Discussion

The objective of this study was to analyze the factors linked to negative age stereotypes in videos created by younger users on TikTok. Findings revealed that these factors include ‘Values and Beliefs of Older Adults’, ‘Negative Encounters with Older Adults’ and ‘Older Adults Antagonizing the Young’. Meanwhile, the depiction of older adults as ‘Warm’ was associated with reduced negative age stereotyping.

In over half of the videos, older adults were stereotyped by younger people as possessing values and beliefs at odds with those of the latter. This echoes past literature which indicates that younger persons tend to view their older counterparts as impeding their more progressive goals related to gender, sexuality and race [3, 22]. By framing their political concerns within the context of generational differences, these younger users assert their own generational consciousness around various social and political issues.

Many videos featured younger individuals reenacting encounters where they were subject to derision by older adults because of their youth. In these reenactments, younger people ranted about how they were frequently typecast as hypersensitive, narcissistic or addicted to technology. Protzko and Schooler [38] contended that certain cognitive mechanisms grant individuals a sense of illusory superiority, which consequently prompts them to disparage the present generation of youth more so than youth from earlier generations. In line with social identity theory [36, 37], negative attitudes towards older adults may function as a defense mechanism on the part of younger individuals.

Negative encounters with older adults significantly predicted negative age stereotyping. Videos on this topic featured younger people attributing the negativity of such encounters to the fact that these people were part of the older cohort. Consistent with intergroup threat theory, prior negative contact with a particular social group can be a key driver of anxiety towards that group [39]. Such negative contact may also intensify feelings of prejudice and the propensity to avoid members of a particular group due to the anticipation that future interactions might likewise be negative [58, 59].

Content portraying older adults as warm reduced the chances of negative age stereotyping. This reiterates past scholarly claims which suggest that older people are generally stereotyped as being high in warmth [57]. In these videos, the term ‘Baby Boomer’ was used more as a demographic label rather than a derogatory term, with younger people admiring older adults for traits such as friendliness and sincerity.

### Practical significance

These findings offer several insights for practitioners. First, more effort should be made to raise public awareness of ageism as a form of prejudice. This is especially critical in view of recent findings unveiling that individuals with egalitarian beliefs—specifically those who champion gender and racial equality—are actually more likely to endorse ageist beliefs [60]. Second, policymakers must pay heed to the fact that unfavorable attitudes towards older adults may stem from a history of negative contact with them. Since the depiction of older adults as being warm lessened the chances of negative stereotyping, it is crucial that younger people are exposed to counter-stereotypical exemplars of older adults, such as those who are more sociable [57]. While stereotypes of warmth may reinforce benevolent ageism [61], they may nevertheless provide a protective buffer against stereotypes of older adults as being antagonistic.

Third, attempts to reframe aging [62–64] as well as to build intergenerational solidarity should be premised on the understanding that ageism cuts both ways. Just as younger people should be mindful not to homogenize older adults as sharing the same values and beliefs, effort should be made to alert older individuals to their own cognitive biases [38] and the effects of these biases on the way they treat younger people. At present, there is limited literature on interventions to tackle ageism against younger people [65]. More research in this area should therefore be conducted to ensure that the needs and interests of younger people are not neglected in the global campaign to combat ageism [66]. Finally, in order not to divert attention from more important questions of power and inequality, the media should steer clear of the notion that certain societal problems, such as climate change or economic strife, can simply be ascribed to any one age group or generation [46]. Rather than sensationalize differences between older and younger groups, the media ought to promote a sense of communality by emphasizing collective goals and cross-generational collaboration [67]. Intergenerational connection will ultimately promote the well-being of both older and younger groups [5].

### Limitations and future directions

This study has several limitations. First, the facial recognition software used to determine users’ ages may have a margin of error [50, 51]. Consequently, some of the videos analyzed may not have been created by users belonging to our desired age range. Attitudes towards older adults could be explored using alternative methods such as interviews, surveys [68–72] and big data analytics [73–81]. Second, most videos were created by individuals from the West. Negative age stereotypes may assume a different form in Asia, where the value of filial piety is generally believed to influence attitudes towards older persons [82–84]. Future research could consider analyzing content from other platforms like Weibo or Douyin, both of which are widely used in China. Third, the videos analyzed represent only a fraction of all videos about older adults on TikTok. The hashtags #Boomer and #OkBoomer were queried because they were widely used on the platform at the time of analysis. Future studies should query other search terms to build a more extensive dataset. Fourth, as we did not have information regarding users’ intentions for uploading the videos, the conclusions drawn were heavily dependent on our coding criteria. To minimize any biases, each video was rated independently by two researchers—a process which yielded high inter-rater reliability.

## Conclusion

With Baby Boomers swelling the ranks of the aging population, it is imperative that society relinquishes its tendency to stereotype individuals on the grounds of age. By examining the possible mechanisms driving negative stereotypes of older adults on TikTok, our study provides the basis upon which such stereotypes can be counteracted. In doing so, it paves the way both to improve the well-being of older persons and to foster intergenerational solidarity.
